# Imaging of muscle and adipose tissue in the spine: A narrative review

**DOI:** 10.1097/MD.0000000000032051

**Published:** 2022-12-09

**Authors:** Fan Yang, Zhengang Liu, Yuhang Zhu, Qingsan Zhu, Boyin Zhang

**Affiliations:** a Department of Orthopaedics, China-Japan Union Hospital of Jilin University, Changchun, China.

**Keywords:** fat infiltration, intraspinal canal fat, lumbar subcutaneous fat, paraspinal muscle, sarcopenia

## Abstract

Interpretation of the morphology and characteristics of soft tissues, such as paravertebral muscles and fat, has always been a “relative blind spot” in the spine. The imaging features of the non-bony structures of the spine have been studied and reinterpreted, and changes in the non-bony structure are associated with spinal disease. Soft tissue parameters such as, the “paraspinal muscle cross-sectional area,” “subcutaneous fat thickness,” and the “paraspinal muscle fat infiltration rate” on computed tomography, magnetic resonance imaging and other imaging techniques are reproducible in the diagnosis, treatment and prognosis of spinal disorders and have the potential for clinical application. In addition, focus on the association between sarcopenia and spinal epidural lipomatosis with spinal disorders is increasing. Currently, there is no summary of studies on fat and muscle in the spinal region. Given this, within the context of recent research trends, this article provides a synthesis of research on adipose and muscle tissue in the spine, discusses advances in the study of the imaging manifestations of these structures in spinal disorders, and expands the perspectives.

## 1. Introduction

In clinical practice, imaging in spinal surgery focuses on bony structures, disc tissue, ligaments, and joint capsule tissue pathology. With the development and functional refinement of ancillary spinal surgical techniques, traditional “blind spots,” such as the paraspinal musculature and fat, have proven to be important in the progression, diagnosis, prognosis, and surgical planning of spinal disease.^[1–4]^ The advantages of muscle morphology based on magnetic resonance imaging (MRI), computed tomography (CT), and musculoskeletal ultrasound have been widely reported, and previous literature has discussed its association with spinal pathology, degenerative spinal pathology, and low back pain.^[5]^ Soft tissue parameters, such as the “cross-sectional area (CSA) of paraspinal muscles” and the “fat infiltration (FI) rate of paraspinal muscles,” on CT, MRI, and other imaging studies can be used to describe paraspinal muscle degeneration, mainly in terms of the reduced cross-sectional area of the paraspinal muscles, reduced muscle density, and increased FI.^[6,7]^ In addition to intramuscular adipose tissue changes, scholars are becoming increasingly aware of the distribution of subcutaneous fat in the spinal canal, which has expanded the boundaries of diagnosis and prognostic assessment of spinal disorders.^[8,9]^ Therefore, in this review, we briefly discuss the progress of research on adipose tissue and paraspinal muscles in the spinal region, aiming to summarize the value and problems of the corresponding imaging changes in the development and clinical management of spinal diseases and to provide a reference for future research.

## 2. Methods

### 2.1. Search strategy

Searches were performed in three electronic databases, PubMed, Embase, and Cochrane, from the start of the databases to January 2022, with the authors focusing on relevant literature from the last 10 years, but also including some published > 10 years ago, as they contain useful information. Our search queries in the three databases were ((((((((((spine) OR (Vertebral Column)) OR (Column, Vertebral)) OR (Vertebral Columns)) OR (Spinal Column)) OR (Column, Spinal)) OR (Columns, Spinal)) OR (Spinal Columns)) OR (Vertebra)) OR (Vertebrae)) AND (((((((fat infiltration[Title/Abstract]) OR (fat thickness[Title/Abstract])) OR (intramuscular fat[Title/Abstract])) OR (intraspinal canal fat[Title/Abstract])) OR (paraspinal muscle[Title/Abstract])) OR (sarcopenia[Title/Abstract])) OR (Spinal Epidural Lipomatosis [Title/Abstract])).

The following inclusion criteria were used for all searches: English language; and All studies on the role of paraspinal muscle and adipose tissue in spinal disease. The following exclusion criteria were used for all searches: Duplicate articles; Literature with unavailable full text or abstracts only; and Literature not related to the research topic.

### 2.2. Study selection

**Figure 1.**
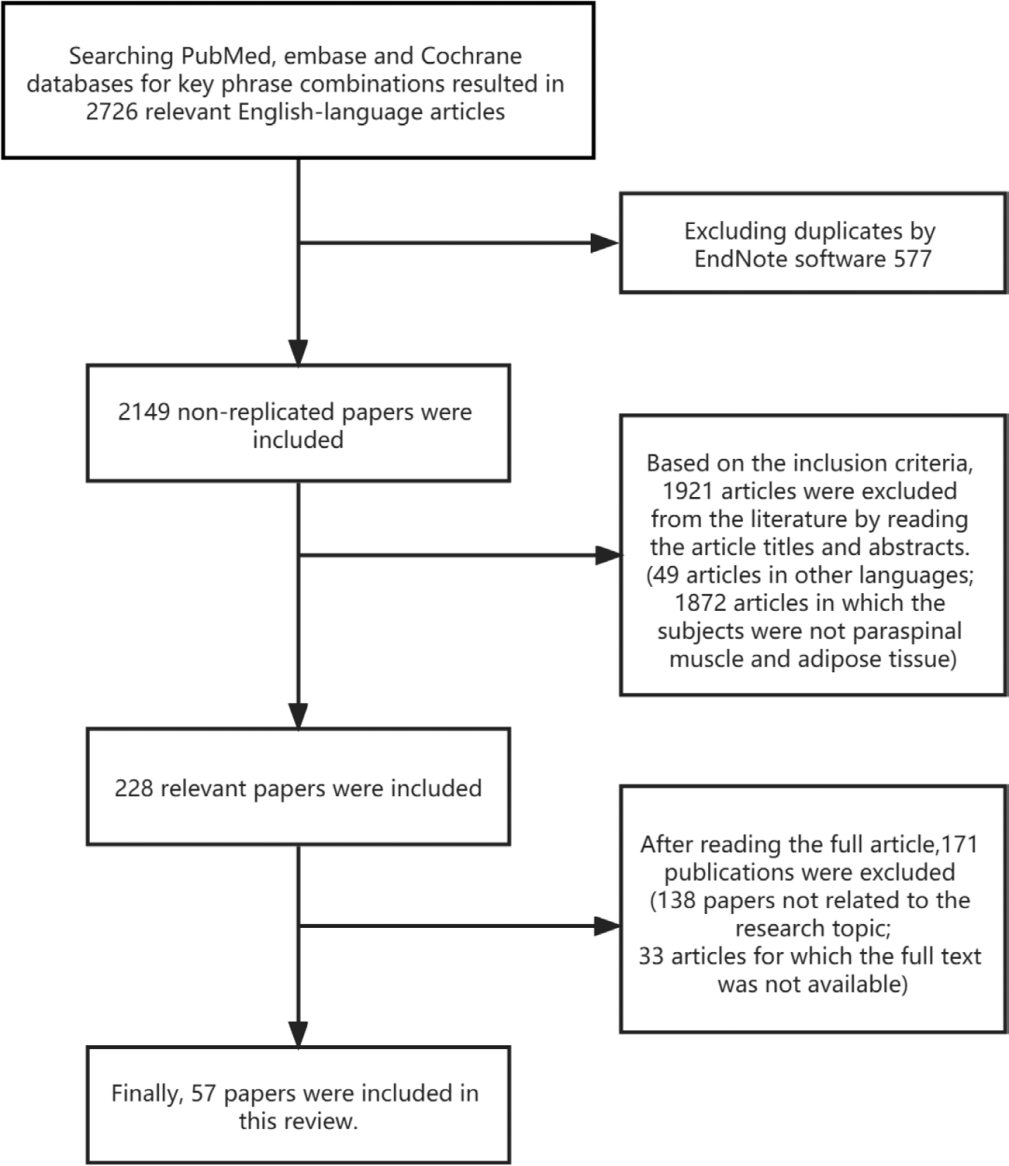
Flow chart of literature screening. A total of 57 papers were finally included.

Two authors (F.Y. and Z.L.) were involved in the literature screening process, in which disagreements arising were resolved through discussion among all authors. First, we found 1656 publications from PubMed, 753 from Embase, and 317 from Cochrane, obtaining a total of 2726 publications. After excluding 577 duplicates through Endnote, a total of 2149 non-duplicates were included. Two independent reviewers then independently screened the literature based on the above inclusion criteria by browsing article titles and abstracts, narrowing the literature to 228 articles. A detailed reading of the full text of the 228 articles excluded 33 articles for which the full text was not available and 138 articles that were not relevant to the study topic. Finally, the authors selected 57 studies for inclusion in the review for discussion. The flow chart is shown in Fig. 1.

As this is a narrative review, no ethical approval was required.

## 3. Spine imaging technology

**Figure 2.**
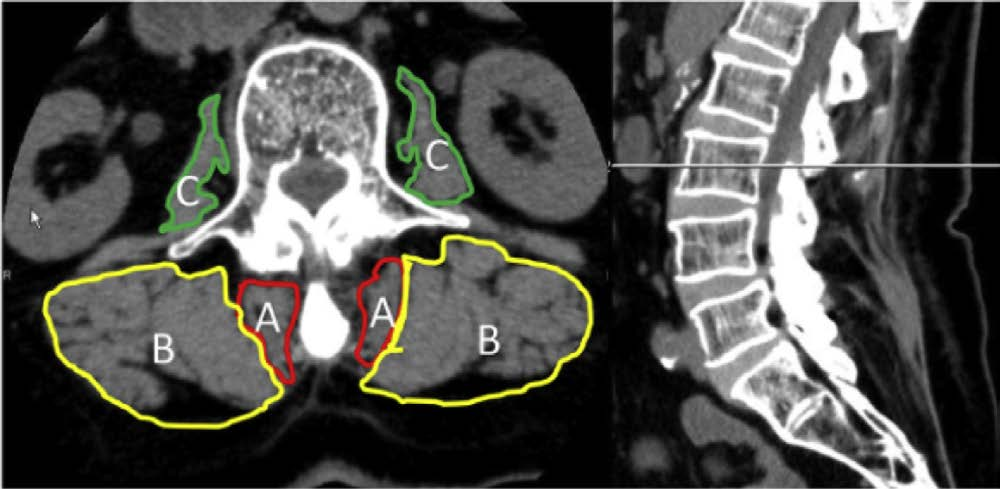
Kameyama et al^16]^ applied the summary method, as proposed by previous authors, to measure the cross-sectional area and muscle density on CT axial images of the paraspinal muscles. A: multifidus; B: erector spinae; C: lumbaris major. (Cited in Ref.^[16]^). CT = computed tomography.

Muscle atrophy and increased fatty infiltration indicate paraspinal muscle degeneration. Muscle atrophy can be subdivided into myogenic and neurogenic. Currently, CT and MRI are the most commonly used techniques to quantify paraspinal muscle atrophy and fatty infiltration.^[10]^ MRI can characterize large differences in the signal intensities of fat and muscle, and intermuscular fat planes are easily imaged, thus separating muscle mass from muscle groups. When muscles degenerate owing to denervation or immobilization, they undergo various histological changes. There are two signs of muscle degeneration that can be easily detected by MRI: a decrease in the volume and deposition of fat and connective tissue.^[7,11]^ It is now known that myogenic and neurogenic atrophy present a high signal on MRI T2-weighted images and a low signal on T1-weighted images and show enhancement after injection of gadolinium-containing contrast agents,^[12]^ whereas Intermuscular fat appears as a high signal area within the paraspinal muscle on T2 axial images, and muscle degeneration can be assessed by calculating the percentage of fat content.^[7]^ Previous studies have shown that CT is equally useful in assessing paraspinal muscle atrophy and fatty infiltration.^[13,14]^ Myogenic atrophy shows reduced muscle density without morphological changes on CT, while neurogenic atrophy shows a reduction in muscle size.^[12]^ Adipose tissue can be easily identified as a localized area of hypodensity on CT scans. In addition, an increase in fatty infiltration is reflected by a decrease in mean density (Hounsfield units (HU))^[15]^ (Table 1 summarizes the pathological manifestations and corresponding imaging features of the paraspinal muscle and fat). Kameyama, et al^[16]^ showed a significant correlation between the fat infiltration rate assessed using MRI and muscle density assessed using CT, and both showed a significant correlation in the assessment of the CSA of the paraspinal muscles. CT is easier to use in the clinical setting because of its simplicity and reproducibility (Fig. 2). Nevertheless, MRI is still recognized as the gold standard for identifying paraspinal muscle changes, and although assessing muscle mass (e.g., fatty infiltration) in clinical practice is often time-consuming and requires an experienced technician, it provides a clearer picture of paraspinal muscle changes and provides more additional information than CT.

**Table 1:** Imaging features of paraspinal muscle pathological manifestations (excluding infections, congenital, trauma, tumor, etc).

Paraspinal muscle pathology		Imaging features	
Muscle atrophy	Myogenic atrophy	MRI： Muscle signal is high on T2-weighted images, low on T1-weighted images, and shows enhancement after gadolinium-containing contrast injection, suggesting muscle edema.^[12]^	CT： CT shows decreased muscle density with no morphological changes.^[12]^
	Neurogenic atrophy	MRI: High signal on T2-weighted image and low signal on T1-weighted image of the affected muscle, with significant enhancement after an intravenous injection of gadolinium contrast agent.^[12]^	CT: CT shows a decrease in muscle size with a contour change, but only a slight decrease in density.^[12]^
Fat infiltration		MRI: The high signal areas within the paraspinal muscles observed on T2 axial images can describe the degree of FI, and muscle degeneration can be assessed by the percentage of fat content in individual muscle CSA.^[7]^	CT: Localized areas of hypodensity are easily identified on CT scans. The degree of fatty infiltration can be more accurately measured by the average density of CT scans (HU), and an increase in fatty infiltration is reflected in a decrease in HU values.^[15]^

CT = computed tomography, CSA = cross-sectional area of paraspinal muscles, FI = fat infiltration, HU = hounsfield units, MRI = magnetic resonance imaging.

## 4. Spinal imaging related to adipose tissue

### 4.1. Intraspinal canal fat and spine disease

**Figure 3.**
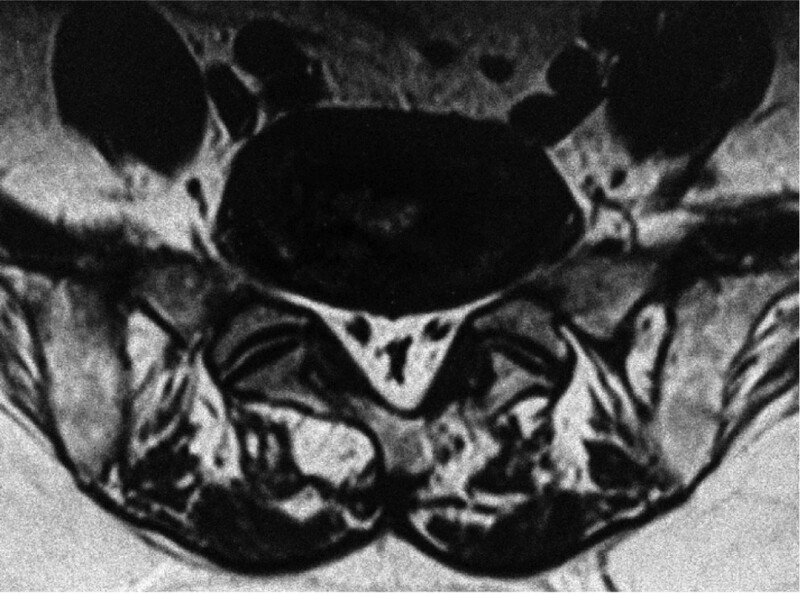
Our hospital treated an elderly male who suffered from neurogenic intermittent claudication. According to MRI imaging (axially) of the lumbar spine, fatty deposits are observed in the L5-S1 spinal canal, and there is a Y-shaped compression of the dural sac.^[19]^ MRI = magnetic resonance imaging.

Spinal epidural lipomatosis (SEL) is a rare disease with a series of neurological symptoms caused by excessive fat deposition in the epidural space of the spinal canal, resulting in spinal cord or nerve root compression. Back pain and lower extremity weakness are the most common symptoms, and some patients have bowel and bladder dysfunction. The possible etiologies include exogenous steroid use, endogenous steroid hormone disease, obesity, surgical induction, and idiopathic disease.^[17]^ CT and MRI are used to diagnose the disease, with MRI being more definitive than CT on the specifics of nerve compression and should be preferred. Nevertheless, there are no clear criteria for the thickness of epidural fat in imaging. In the early years, Lee et al^[18]^ proposed that the diagnosis can be confirmed when the thickness of the epidural fat layer is greater than 7 mm. Additionally, Sugaya et al^[19]^ found that the dural sac would be compressed, and the “Y” sign appeared when epidural fat was increased by analyzing SEL MRI data (Fig. 3). Although MRI can be used to observe the shape and location of epidural adipose tissue, most doctors still focus on structure-induced nerve compression, which leads to occasional misdiagnosis and missed diagnosis of SEL. In addition, we believe that the patient’s extensive medical history, presence of mature adipose tissue, and lack of tendency for malignant transformation are obstacles that hinder doctors from fully evaluating the disease progression and neurological prognosis of patients with SEL. Hence, in clinical practice, the possibility of SEL needs to be considered and differentiated based on MRI examinations when recalcitrant low back pain or neurological symptoms are found in such populations. Borré et al^[20]^ classified excessive deposition of epidural fat in the lumbar spine as grade 0 to III based on the ratio of the anterior-posterior length of the epidural fat to the anterior-posterior length of the spinal canal (EF/SC-L), as revealed by MRI, and suggested that when this ratio exceeds 75% (i.e., grade III), there is a high probability of neurogenic claudication with cauda equina syndrome. Recently, Sasagasako et al^[21]^ proposed a new grading system based on EF/SC-L ≤ 50% for grade I, EF/SC-L of 51% to 74% for grade II, and EF/SC-L ≥ 75% for grade III, and found that decompression surgery in grade I and II patients did not achieve the desired outcome. Therefore, the treatment options for SEL remain highly controversial, but conservative treatment remains the preferred option for acute neurological dysfunction (e.g., paresis, cauda equina syndrome). Therefore, the diagnostic and treatment plan should clarify its etiology, so direct surgical intervention may not be the best option.

### 4.2. Lumbar subcutaneous fat and spine disease

Adipose tissue is an important structure around the spine, often found subcutaneously, in the paravertebral muscles, vertebral bodies, and the spinal canal. Previous studies have focused less on the imaging features of peri-spinal adipose tissue. Compared to CT, MRI is more efficient in distinguishing different soft tissues because MRI has two different imaging characteristics (T1-weighted and T2-weighted) and no radiation handling capabilities, which allow a clear picture of the morphology and distribution of fatty tissues around the spine.^[13]^ Therefore, it has high accuracy and obvious advantages in quantifying the fat content of muscles. The current consensus is that axial T2-weighted vertical distance from the tip of the spinous process to the skin on MRI of the lumbar spine can be used to measure lumbar subcutaneous fat thickness.^[14]^ Odeh et al^[9]^ found that an increase in posterior lumbar subcutaneous fat thickness was an independent risk factor for surgical area infection compared with BMI. For every 1 mm increase in fat layer thickness, the odds of infection increased by approximately 4.9%. In addition, Lee et al^[14]^ found a 4-fold increase in surgical site infections in patients with a subcutaneous fat thickness greater than 5 cm at L4. Shaw et al^[22]^ further defined the ratio of subcutaneous fat thickness to spinous process height in the posterior lumbar surgical area as the subcutaneous lumbar spine index (SLS). SLS was measured in 285 patients after lumbar surgery using MRI, and it was found that SLS was closely correlated with the occurrence of postoperative complications after lumbar surgery. There is a considerable increase in the rate of perioperative complications, such as fat liquefaction, when SLS is greater than 5.8, and SLS is greater than 3.81 indicates the possibility of revision after surgery. Thus, fat distribution in the posterior lumbar surgical area has a predictive role in the efficacy of surgery and occurrence of complications. We believe that the predictive effect of a high SLS score on surgical complications may be due to the following reasons. First, open spine surgery requires clear exposure to the surgical area, and excessive adipose tissue may worsen soft tissue injury and prolong the exposure time. Second, a high-frequency electric knife generates a high temperature, which leads to fat liquefaction. A thicker subcutaneous fat layer significantly increases the probability of factual liquefaction and delays timely detection of fat liquefaction. Additionally, obese patients are more likely to have underlying diseases such as diabetes and hypertension. Therefore, for obese patients, perioperative preparation should be performed to fully consider adipose and soft tissue factors and evaluate adverse prognostic factors related to fat parameters. The thickness of subcutaneous adipose tissue in the upper lumbar spine is also a significant predictor of intervertebral degeneration and Modic changes in the lower lumbar spine.^[13]^ The results thus far suggest that subcutaneous fat in the lumbar spine is associated with postoperative complications and has some value in predicting pathological changes in spinal diseases; therefore, reducing the thickness of subcutaneous fat may prevent the occurrence of spinal diseases.

## 5. Spinal imaging related to the paraspinal muscles

### 5.1. Paraspinal muscles and cervical disease

Neck pain is one of the most common causes of disability, followed by back pain, depression, and musculoskeletal disorders.^[23]^ Approximately 5.9% to 38.7% of adults experience varying degrees of neck pain.^[24]^ Similar to the pathogenesis of lower back pain, the causes of cervical back pain are correlated with pathological changes in the intervertebral disc, vertebral body, and neck muscles, which are primarily manifested in cervical spondylosis. In patients with chronic nonspecific neck pain, studies have been unable to draw definitive inferences from MRI findings of paraspinal muscles in patients with chronic nonspecific pain.^[25]^ However, Van Looveren et al^[26]^ hypothesized that muscle atrophy in patients with chronic neck pain is an avoidance response to pain. In addition to factors such as disc degeneration, recent studies have revealed that pathological changes in the cervical paraspinal muscles play an important role in the development and progression of cervical spondylosis.^[27]^ Bone structure, intervertebral discs, and ligaments are the foundation of cervical spine stability, and muscles are the supportive structures that ensure the movement and stability of the cervical vertebra; therefore, its atrophy will inevitably cause instability of dynamic balance, leading to cervical stress and stability changes and accelerating cervical degeneration. This may explain why the degeneration of the paraspinal muscles accelerates the occurrence and development of cervical spondylosis. Some studies have reported that the degree of fat infiltration in the paraspinal muscles of patients with ossification of the posterior longitudinal ligament (OPLL) of the cervical spine is significantly associated with lesion size and ossification encroachment rate. Furthermore, the neck disability index score and fat infiltration rate were significantly higher in patients with continuous or mixed OPLL than in those with focal OPLL.^[28]^ Function of the paraspinal muscle group is also associated with postoperative complications. Usami et al^[29]^ evaluated the swelling of the multifidus muscle in the early postoperative period using magnetic resonance imaging and found that the gradual swelling of the multifidus muscle was accompanied by a gradual increase in nerve traction, which resulted in the development of C5 nerve root palsy. Thus, a relatively small intraoperative disruption of the posterior cervical muscles may prevent the onset of C5 nerve root palsy. Perhaps other muscles may also influence the onset of C5 nerve palsy by the same mechanism as the multifidus, which still needs to be confirmed in future retrospective clinical studies.

### 5.2. Paraspinal muscles and lumbar disease

**Figure 4.**
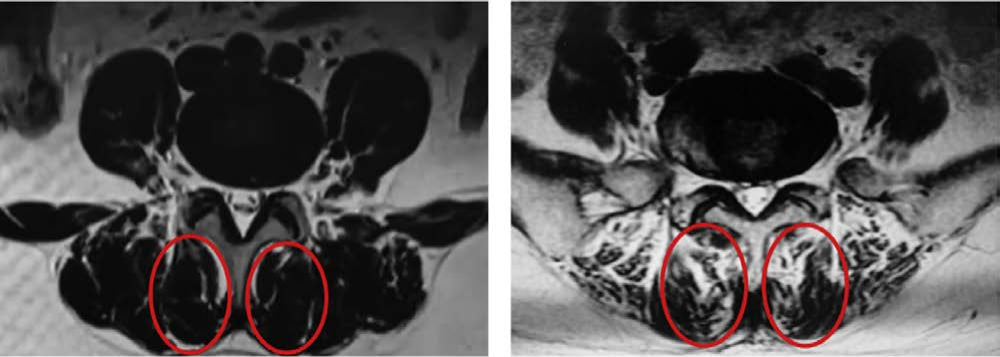
Examples of different levels of fat infiltration in the multifidus muscle. The area in the solid line on the left shows multifidus fat volume < 50%, and the area in the solid line on the right shows multifidus fat volume > 50%. When the percentage of multifidus FI is > 50%, the patient has a significantly higher risk of low back pain and mobility disorders.^[34]^FI = fat infiltration.

Currently, the initial diagnosis of low back pain relies heavily on the patient’s chief complaints. Muscle dysfunction and morphological changes, including decreased muscle strength, endurance, increased fat content, and changes in muscle volume, are commonly reported in patients with low back pain (LBP) compared to the asymptomatic population.^[11,30]^ These changes in muscle function and structure are the main features of paraspinal muscle atrophy. FI of paraspinal muscles is one of the important signs of degeneration of paraspinal muscles, and its essence is the replacement of muscle fibers by adipose or non-contractile connective tissue.^[31]^ Fat infiltration in muscles reduces the proportion of force-producing contractile tissue, thus affecting the function of these important muscles in the lumbar spine.^[32]^ The fatty infiltration of the paraspinal muscles increased significantly with age. At the early stage of paraspinal muscle degeneration, although fat replacement occurs in the paraspinal muscles, there is usually no significant change in the CSA of the paraspinal muscles at this stage because of the space-occupying effect of fat. Therefore, FI can sensitively reflect degeneration of the lumbar and paraspinal muscles. Researchers have studied the paraspinal muscles using MRI scans and magnetic resonance spectroscopy and found that the distribution of fat in the paraspinal muscles is related to low back pain. Teichtahl et al^[33]^ found that fat infiltration in the multifidus was more common than in other paraspinal muscles by quantitative measurement of FI in the paraspinal muscles of patients with low back pain. When the proportion of FI of the multifidus was greater than 50%, the risk of low back pain and lumbar movement disorders was significantly increased (Fig. 4). At the same time, they also found that the severe FI phenomenon of the multifidus was significantly correlated with lumbar Modic type II degeneration and intervertebral height loss. Similar degenerative changes in the paraspinal muscles have also been observed in patients with neck pain. Kim et al^[34]^ found that increased fat infiltration in the upper cervical dorsal muscle was significantly associated with loss of cervical physiological pronation, which reflected higher VAS scores, whereas greater rates of fat infiltration in the lower cervical dorsal muscle were associated with poorer neck motion and stability function. In an analysis of the proton density fat fraction and bone mineral density (BMD) of paraspinal muscles in 88 participants using chemical shift encoding-based water-fat MRI and quantitative CT, Zhao et al^[35]^ reported that the proton density fat fraction of the paraspinal muscles was significantly increased in those with decreased BMD, suggesting that the phenomenon of lumbar FI was negatively correlated with BMD. Hence, increased paraspinal muscle fat infiltration can be used as an imaging marker to identify patients with low BMD, and is also expected to help predict the risk of cancellous fractures.

The cross-sectional area of the paraspinal muscles can accurately reflect the changes in muscle volume and morphology in each group of muscles, which is one of the important parameters for evaluating paraspinal muscle atrophy.^[36]^ Some investigators have measured and compared the groups within the paraspinal muscles using MRI and found that the CSA of the multifidus in patients with low back pain is significantly reduced when compared with healthy adults, with a mean difference of approximately 10%. For patients with unilateral nonspecific low back pain, the CSA of the multifidus on the painful side is significantly smaller than that of the unaffected side, and the CSA of other paraspinal muscles usually lacks significant changes when compared with multifidus atrophy in patients with low back pain.^[7]^ Therefore, the reduction in CSA in the multifidus is expected to be used as an objective basis for diagnosing chronic low back pain. Nevertheless, the diagnostic value of CSA of the paraspinal muscles for low back pain remains controversial. This is mainly because the correlation between paraspinal muscle atrophy and low back pain is affected by several factors. Changes in age, posture, and body position may affect the CSA measurement results. Paraspinal muscle edema can persist for up to 6 to 10 months after surgery, and the observation and measurement of CSA, as well as the extent of muscle atrophy during this period, cannot be eliminated based on MRI fat suppression techniques.^[37–39]^ Endplate inflammation and its related modulatory changes are common pathological factors of chronic low back pain. Recently, it has been found that paraspinal muscle atrophy and fat infiltration are significantly higher in patients with Modic type I/II changes than in the healthy population.^[40]^

Spine degeneration is a complex process that includes skeletal, neural, and muscular degeneration.^[41,42]^ It is generally accepted that degeneration of the vertebral body and intervertebral disc plays a key role in this process. However, as an important structure for maintaining spinal stability, the degeneration and atrophy of various components in the paraspinal muscle group aggravate the instability of adjacent discs, facet joints, and ligaments, which leads to a decrease in the overall stability of the spine. In recent years, the significance of paraspinal myopathy in the development of spinal degeneration has gradually been emphasized, providing new insights into the diagnosis and treatment of spinal degeneration. Degenerative lumbar scoliosis (DLS) is the most common spinal deformity observed in adults. As effectors of somatic postural reflexes, the paraspinal muscles are involved in maintaining trunk posture and the regulation of spinal curvature, and the extensor muscle group plays a more important role in antagonizing the development of DLS than the flexor muscle group. Studies have shown that the volume of paraspinal muscles in patients with DLS is significantly reduced in the scoliosis segment, and this change is more evident on the concave side of the scoliosis segment. The degree of asymmetric change in the paraspinal muscles is positively correlated with variables such as the Cobb angle, parietal rotation, and lumbar translation because these variables change the load of the paraspinal muscles, which affects the stability of the spine.^[43]^ Thus, the paraspinal muscles have a significant compensatory motivation for scoliosis, and the degree of this asymmetric change is suggestive of scoliotic progression and deterioration. Tang et al^[44]^ found that by analyzing scores of the SF-36 and ODI for lumbar function and quality of life in 49 patients with DLS, the quality of life in patients with DLS was significantly reduced when the CSA of the multifidus decreased. Degenerative spondylolisthesis is mainly due to lumbar instability after degeneration. In addition to the degenerative factors of the intervertebral disc and facet joints, studies have found that the paraspinal muscles play an essential role in the development of the disease. Wang et al^[45]^ found that the CSA of the multifidus at the slipped level was significantly reduced in patients with lumbar degenerative spondylolisthesis, but the corresponding erector spinae became hypertrophic and the CSA was significantly increased. In terms of pathogenesis, ES hypertrophy may also have a compensatory effect on spinal instability and multifidus atrophy. Furthermore, the degree of paraspinal muscle atrophy positively correlates with spondylolisthesis severity of spondylolisthesis.^[2]^ Therefore, these findings may provide relevant preventive exercise programs and interventions for degenerative disease.

### 5.3. Sarcopenia and spine disease

The term myasthenia gravis was first coined by Rosenberg in 1989.^[46]^ Unlike muscle degeneration, which results in muscle atrophy due to increased fatty infiltration, and although the presence of these pathological changes is often characteristic, its manifestation cannot be defined as sarcopenia. Sarcopenia is an age-related skeletal muscle dysfunction characterized by progressive loss of muscle volume and weakness of muscle strength. Although the pathogenesis remains unclear, relevant studies have suggested that the occurrence of sarcopenia is correlated with factors such as osteopenia, limb motor disability, poor nutrition, neuromuscular junction abnormalities, and mitochondrial dysfunction.^[47,48]^ The occurrence and development of sarcopenia and cervical and lumbar spine diseases are often mutually causal factors. As an important participant in spinal movement, loss of muscle volume in the paraspinal and abdominal muscles directly affects spinal function. Spinal motor dysfunction may further increase muscle disability. Wu et al^[48]^ found that the incidence of sarcopenia is approximately 24.8% in patients with lumbar degeneration. Matsuo et al^[47]^ reported that the paraspinal muscle volume was significantly reduced in patients with sarcopenia by studying 178 patients with lumbar spinal stenosis. Additionally, the incidence of low back pain, lumbar spondylolisthesis, and pelvic imbalance in the sarcopenia group was much higher than that in the control group. In elderly patients undergoing lumbar surgery, the degree of muscle volume loss is positively correlated with the degree of postoperative pelvic imbalance. Moreover, sarcopenia and fat infiltration in the paraspinal muscles are considered risk factors for postoperative lumbosacral deformities.^[49]^ For patients with sarcopenia, deterioration of postoperative muscle dysfunction caused by surgical dissection and postoperative muscle scarring may be important causes of adverse events. Furthermore, muscle satellite cell reduction in skeletal muscle severely constricts the muscle regeneration capacity after injury. Therefore, preoperative evaluation of paraspinal muscle sarcopenia is crucial for predicting postoperative complications and determining surgical prognosis.

### 5.4. Effect of paraspinal muscle status on surgical outcome

Previous studies have quantified paraspinal muscles based on MRI or CT images using specific software or methods and found that paraspinal muscle atrophy is more obvious after lumbar fusion than after decompression alone.^[50,51]^ However, significant postoperative paraspinal muscle atrophy may hinder long-term postoperative performance and recovery.^[52]^ However, studies have found that paraspinal muscle volume decreased postoperatively, even with anterior stand-alone fusion.^[53]^ The results suggest that lumbar fusion is the direct cause of postoperative paraspinal muscle atrophy and that the surgical approach has little effect on the condition. Leng et al^[54]^ studied parameters, such as the CSA of paraspinal muscles in 137 DLS patients who underwent corrective surgery and found that for patients with more than six fusion levels, the proportion of functional paraspinal muscle less than 0.71 (functional cross-sectional area/total cross-sectional area of erector spinae) was an independent risk factor for postoperative lower vertebral fixation loosening. Patients with lower lumbar muscularity are at a higher risk of proximal junctional kyphosis after long-level correction.^[55]^ Sarcopenia is an important factor affecting the efficacy of lumbar surgery and postoperative quality of life, an independent risk factor for the occurrence of surgical adverse events, especially psoas major sarcopenia, and an independent risk factor for the occurrence of proximal junctional kyphosis and proximal junctional failure after spinal correction.^[56]^ Koshimizu et al^[57]^ reported that the risk of sagittal cervical imbalance after posterior cervical laminoplasty was much greater in patients with sarcopenia than in the controls. Thus, the status of paraspinal muscle degeneration is important for evaluating surgical outcomes and determining the surgical prognosis. Targeted paraspinal muscle exercises may be beneficial in improving surgical outcomes in patients with a higher risk of surgical failure.

## 6. Conclusions

MRI is the examination modality of choice for interpreting muscle and adipose tissue changes. The effective interpretation of these soft tissue structures not only covers the traditional “blind spots” in the interpretation of imaging examinations, but also provides a more comprehensive clinical view for the spine surgeon.

First, nerve compression by intradural fat is easily overlooked, distinguished from traditional neurocompressors, and requires attention. Second, subcutaneous fat thickness and SLS index have been shown to be predictive cues for postoperative spinal complications, and their clinical application is undeniable.

Second, unlike adipose tissue, the paraspinal muscle is more significant in assessing spinal disorders; for example, the application of indices, such as paraspinal muscle CSA and FI, have significant clinical value. Even so, we identified significant heterogeneity in the research methods, which may be explained by the morphology and function of the paraspinal muscles in relation to the wider spinal field. Therefore, the indices for evaluating the paraspinal muscles still need to be refined and standardized.

## Author contributions

**Conceptualization:** Yuhang Zhu.

**Supervision:** Qingsan Zhu.

**Visualization:** Fan Yang.

**Writing – original draft:** Fan Yang, Zhengang Liu.

**Writing – review & editing:** Boyin Zhang.
